# Working With Underachieving Students in Higher Education: Fostering Inclusion Through Narration and Reflexivity

**DOI:** 10.5964/ejop.v12i4.1321

**Published:** 2016-11-18

**Authors:** Laura Nota

**Affiliations:** a University of Padova, Padova, Italy

[Other p1]The book *Working With Underachieving Students in Higher Education: Fostering Inclusion Through Narration and Reflexivity* (2016) edited by Maria Francesca Freda, José González-Monteagudo, and Giovanna Esposito, is a very interesting and innovative work for those who wish to engage in a critical reading of the relation between academic inclusion, narration and reflexivity. The book stems from a European action-research project, INSTALL (Innovative Solutions to Acquire Learning to Learn), which was founded in 2011 by the European Commission within the ERASMUS Multilateral Project measures. It presents an innovative narrative methodology, the Narrative Mediation Path (NMP), which is aimed to foster reflexive competences and to improve the academic performance of underachieving college students at risk of drop-out and university exclusion.

The book is divided into three sections. The first part (Chapters 1–4) presents key issues in the research (reflexivity, academic inclusion, and narrative) that formed the basis of the theoretical and methodological approaches in the INSTALL Project. Specifically, this section aims to present an innovative model of academic inclusion and offer the theoretical background to reflexivity and narration. Moreover it discusses the need of promoting academic inclusion for students at risk of dropping-out, such as non-traditional, disadvantaged and underachieving students. The second part (Chapters 5–10) is devoted to the Narrative Mediation Path, the most significant phases of the training process in the four participating European countries (Italy, Spain, Ireland, and Romania) and its main products from a cross-cultural perspective. Finally, the third part (Chapters 11-12) concerns the implications of the NMP training program for policy and formative practice, by providing suggestions about the need to adopt reflexive training and narrative methods within the higher education (HE) field.

As a reader and a researcher in the field of counselling for students at risk to be excluded, my attention drew to some points of interest that make *Working With Underachieving Students in Higher Education: Fostering Inclusion Through Narration and Reflexivity* a very useful book for many audiences– researchers, professionals, policy makers.

First, the book presents an innovative model of *Academic Inclusion* which focuses on the educational context resources that institutions have to provide to all students, regardless of their backgrounds; moreover, it puts the spotlight on the need to improve the students' resilience, namely the competence in recognizing and using the resources available. Furthermore, I found interesting the idea that inclusion requires both reflection and resilience: to be included, students need to reflect on their position in the university context and to be aware of their personal needs and desires; at the same time, students need to develop resilience, in order to find the necessary resources and use them strategically to achieve their learning goals. And for doing so, the university context has to promote these capacities, in particular to the most vulnerable students.

Second, the book introduces a semiotic and psychodynamic conceptualization of reflexive processes by considering the multidimensional and intersubjective nature of this construct. Nowadays, many scholars (e.g., Burkitt, 2012) agree that there are no unique reflexive processes, instead there are diverse levels of reflexivity with differing complexities. The book provides an overview of the reflexivity construct as it has been addressed in various humanities disciplines (e.g., psychology, pedagogy, and sociology) to highlight points of differences and connections. Moreover, the book conceptualizes reflexivity as a process that cannot be confined to individual minds but is the product of the intersubjective and dynamic exchange between subjects who share the same psychological context. This conceptualization constitutes an important shift of focus, from an idea of reflexivity as an individual and a-contextualized psychological function to the idea of reflexivity as a collective product that assumes different configurations due to the relation between subject and context. Moreover, reflexivity has to be conceived as a *tool* (not the aim) of an intervention. In so doing, reflexive awareness can lead to the implementation of actions appropriate for the context. This is of particular interest in the HE field: at university, reflexivity development can allow students to assess a situation, and take appropriate actions to meet their developmental and learning aims. In this regard, I found particular interesting the development of a multi-dimensional evaluation model to measure both improvements in the reflexive competence of participating students, as well as the relationships between reflexive competence, agency, and academic performance. As stated in the book, agency is a complex construct that refers not only to the set of possible actions implemented in a specific context, but also to actions, decisions, choices and behaviors related to other and all aspects of an individual's life (Caston, 2011). The authors developed an evaluation model that includes indicators for assessing the "objective change" in terms of improvements in the academic performance (GPS, ECTS, number of examinations passed, etc.), as well as some self-perception measures to assess the development of "agentic" dimensions (e.g., relational competences, self-esteem, etc.) and to evaluate the students' self-perception of change. In other words, the model stems from the hypothesis that it is important to understand not only if change occurred in the academic field, but to analyze if the students have also implemented different agentive actions and become *self-authors* (Baxter Magolda, 2009) of their lives and university experience.

Moreover, my attention was drawn to the methodology, the Narrative Mediation Path (NMP), developed and implemented in order to foster reflexivity with underachieving university students. From my perspective, NMP allows students to deeply reflect on their university experiences by providing, on the one hand, a multimodal narrative methodology based on metaphors, vignettes, written narratives and sculpture, and on the other, by using the group as a narrative device to amplify the reflexive processes. The value of narrative methodologies has been widely recognized in the clinical/psychotherapeutic (e.g., Angus & McLeod, 2004), as well as in the counselling field (Savickas, 2005). Nevertheless, I found highly original the adoption of a multimodal narrative approach which seeks to promote personal resources and reflexive competences using four narrative modes (metaphoric, iconographic, writing, and the bodily) and related narrative media (proverbs/mottos, vignettes, written narratives, sculpture). According to the NMP model, the narrative multimodality approach can strengthen and amplify reflexive processes by providing different "reflexive mirrors" which allow students to "return" on their university experience and give a new meaning to it (Esposito & Freda, 2016). The NMP approach is widely discussed in the second part of the book that presents a broad overview of various qualitative methods and tools capable of handling the complexity of meaning-making processes in the narratives of students at risk of dropping-out.

The book is also a rich source of cases illustrating research using some qualitative narrative methods, such as the analysis of narrative functions (Ochs & Capps, 2001), idiographic approaches to the study of reflexive processes (Salvatore & Valsiner, 2010) and the Interpretative Phenomenological Analysis (IPA) (Smith & Osborn, 2003). Readers will find it very useful to read and reflect on these concrete experiences collected with the help of multiple qualitative methods within narrative analysis. The analytic description of the training experiences conducted in different European countries also constitutes a resource for many HE professionals (counsellors, educators, tutors) who constantly have to deal with the difficulties intrinsic in their professional tasks and who need practical guidelines to work with students at risk of exclusion and to evaluate the effectiveness of their work.

Finally, I appreciated the importance the book gives to the implications of this research for HE polices and institutions. As an academic, I am constantly facing the difficulties that universities encounter in this time of crisis that is characterized by the increasing globalization, diversity, complexity, uncertainty, and competitiveness. There is a growing need to develop policy and good practices centred on students at risk of being excluded, such as non-traditional, disadvantaged, and underachieving students who need appropriate learning environments and innovative interventions. The INSTALL project described in this book offers, from my perspective, an interesting approach to develop innovation in education and training by focusing, on the one hand, on the role of international cooperation and collaboration between institutions, and on the other, on the voices and experiences of students through the use of narratives. For this reason, I think that this book is potentially useful to policy makers and HE staff developing narrative approaches in research, intervention, student psychological support, tutoring, teaching, and working with small groups.

In conclusion, *Working With Underachieving Students in Higher Education: Fostering Inclusion Through Narration and Reflexivity* gives us an important opportunity to consider carefully students at risk of university drop-out and exclusion and learn about possible activities and interventions to increase the quality of life for all. I consider this book interesting, capable of cutting across different disciplines with ease but without ever slipping into banality or speculation. It deserves to be read not only for its innovative narrative methods and techniques of intervention but also, beyond the specific target group, because it offers theoretical insights, methodological criteria, and practical techniques for working with higher education students.
